# 两性离子双功能化超亲水金属有机框架纳米复合材料的制备及其用于糖肽的选择性富集

**DOI:** 10.3724/SP.J.1123.2020.11006

**Published:** 2021-03-08

**Authors:** Dapeng LI, Guangshan XIE, Peisi XIE, Lin ZHU, Zongwei CAI

**Affiliations:** 香港浸会大学, 环境与生物分析国家重点实验室, 香港 999077; State Key Laboratory of Environmental and Biological Analysis, Hong Kong Baptist University, Hong Kong 999077, China; 香港浸会大学, 环境与生物分析国家重点实验室, 香港 999077; State Key Laboratory of Environmental and Biological Analysis, Hong Kong Baptist University, Hong Kong 999077, China; 香港浸会大学, 环境与生物分析国家重点实验室, 香港 999077; State Key Laboratory of Environmental and Biological Analysis, Hong Kong Baptist University, Hong Kong 999077, China; 香港浸会大学, 环境与生物分析国家重点实验室, 香港 999077; State Key Laboratory of Environmental and Biological Analysis, Hong Kong Baptist University, Hong Kong 999077, China; 香港浸会大学, 环境与生物分析国家重点实验室, 香港 999077; State Key Laboratory of Environmental and Biological Analysis, Hong Kong Baptist University, Hong Kong 999077, China

**Keywords:** 金属有机框架, 亲水相互作用色谱, 基质辅助激光解吸电离-飞行时间质谱, 选择性富集, 翻译后修饰, 糖肽, metal-organic framework (MOF), hydrophilic interaction liquid chromatography (HILIC), matrix-assisted laser desorption ionization time-of-flight-mass spectrometry (MALDI-TOF MS), selective enrichment, post-translational modification (PTM), glycopeptide

## Abstract

蛋白糖基化是生物体中普遍发生且重要的生物学过程,其参与多种分子生物学的功能和途径,是临床诊断重要的生物标志物。但是,糖肽因其丰度低、离子化效率低、糖链异质性等难点,使糖蛋白分析一直面临巨大的挑战。因此,研究合成了一种新型的两性离子双功能化纳米金(AuGC)修饰的超亲水性沸石咪唑骨架(ZIF-8)纳米复合材料(AuGC/ZIF-8),并建立了亲水相互作用色谱(HILIC)和基质辅助激光解吸电离-飞行时间质谱(MALDI-TOF MS)联用选择性富集糖肽的分析方法。谷胱甘肽和半胱氨酸双功能化的协同作用,使MOF具有超亲水性和低空间位阻,为糖肽选择性富集提供了更多的亲和位点。研究以辣根过氧化物酶(HRP)为模式糖蛋白,通过AuGC/ZIF-8富集后,MALDI-TOF MS分析。结果表明,AuGC/ZIF-8对HRP糖肽的富集能力高达250 μg/mg,且在与牛血清白蛋白(BSA)混合溶液中(HRP-BSA (1∶200,质量比))显示出对HRP糖肽的高选择性,以及极低含量下(0.3 ng/μL)的高灵敏度。因此,在复杂生物样品糖蛋白的富集分离中具有很大的应用潜力。

## Abstract (en)

Protein glycosylation is a ubiquitous and important biological process involved in various molecular functions and biological pathways. It also yields important biomarkers for clinical diagnoses. However, glycopeptide analysis is challenging due to low abundance, low ionization efficiency, and glycan heterogeneity. In the present study, a method based on hydrophilic interaction liquid chromatography (HILIC) was developed for the selective enrichment of glycopeptides using a novel metal-organic framework (MOF) nanocomposite (AuGC/ZIF-8). Dual functionalization with glutathione and cysteine has resulted in an ultra-hydrophilic MOF, with synergistic effects and lower steric hindrance, providing more affinity sites for the glycopeptide enrichment. Horseradish peroxidase (HRP) was used as a model glycoprotein, and AuGC/ZIF-8 was used to enrich glycopeptides prior to analysis by matrix-assisted laser desorption ionization time-of-flight mass spectrometry (MALDI-TOF MS). AuGC/ZIF-8 displayed outstanding performance at enriching HRP glycopeptides, with high enrichment capacity (250 μg/mg), high selectivity in mixtures containing bovine serum albumin (BSA) (HRP-BSA (1∶200, mass ratio)), and high sensitivity at very low content (0.3 ng/μL). Thus this MOF holds promise for in-depth, comprehensive glycoproteomic and related analysis.

蛋白质糖基化是生物体内最丰富且最重要的蛋白质翻译后修饰(PTM)之一,其在蛋白质折叠、稳定、信号转导和免疫应答等生物功能中有着重要作用^[1,2]^。研究表明,异常的蛋白糖基化与许多疾病的发生发展密切相关^[3]^,例如癌症^[4]^、老年痴呆症^[5]^和免疫缺陷^[6]^。因此,糖蛋白组学分析对于深入了解疾病的机理,以及发现生物标志物和药物靶标至关重要^[7,8]^。质谱(MS)一直是糖蛋白组学分析强有力的工具^[9,10]^。然而,糖肽由于丰度低、离子化效率低,且容易受非糖肽信号的干扰,使得基于质谱的糖蛋白组学分析仍然具有巨大挑战^[11]^。因此,在质谱分析前对糖肽进行高效富集,是糖蛋白分析至关重要的一步。

目前,糖肽富集的方法主要包括凝集素亲和法^[12]^、肼化学法^[13]^、硼酸亲和色谱法^[14]^和亲水相互作用色谱法(HILIC)^[15]^。其中,基于HILIC的富集方法因其无偏向性、优异的相容性、良好的选择性和可重复性的优点得到了广泛发展^[16]^。金属有机框架(MOF)纳米材料,由于具有较大的比表面积和均匀且可调控的孔隙^[17]^,目前广泛用于蛋白质组学和糖蛋白质组学分析,例如MIL-101(NH_2_)@Au-Cys^[18]^、UiO-66-COOH^[19]^、Fe_3_O_4_@PDA@Zr-SO_3_H^[20]^等,展现出巨大的应用潜力。因此,开发基于HILIC的亲水性好、富集能力高、制备方法简便的新型MOF纳米复合材料十分必要。

本研究利用两步合成法,制备了一种新型的两性离子双功能化纳米金(AuGC)修饰的超亲水性沸石咪唑骨架(ZIF-8)纳米复合材料(AuGC/ZIF-8),同时联用基质辅助激光解吸电离飞行时间质谱(MALDI-TOF MS),用于糖肽的高效富集和分析。谷胱甘肽(GSH)和半胱氨酸(Cys)双重功能化不仅可以控制和稳定金纳米簇,而且还提供了丰富的游离羧酸和氨基。利用GSH和Cys二者的超亲水性以及低位阻协同作用,实现高效的糖肽选择性富集。利用上述优势,AuGC/ZIF-8复合纳米材料显示出优异的亲水性,良好的生物相容性和稳定性,且对糖肽具有高选择性。

## 1 实验部分

### 1.1 仪器、试剂与材料

粉末X射线衍射仪(XRD)、Rapiflex MALDI-TOF MS仪(Bruker Daltonics公司,德国); X射线光电子能谱仪(XPS, Thermo Electron公司,美国);傅立叶变换红外光谱仪(FT-IR, Perkin Elmer公司,美国)。

六水合硝酸锌(Zn(NO_3_)_2_·6H_2_O)、2-甲基咪唑(2-MIM)、氯金酸(HAuCl_4_)、Cys、GSH、2,5-二羟基苯甲酸(DHB)、碳酸氢铵(NH_4_HCO_3_)、甲酸(FA)、辣根过氧化物酶(HRP)、牛血清白蛋白(BSA)、胰蛋白酶、二硫苏糖醇(DTT)、碘乙酰胺(IAA)均购自美国Sigma-Aldrich公司。

实验过程中使用的水均由Milli-Q超纯水系统纯化去离子水(Millipore公司,美国)。HPLC级乙腈(ACN)和甲醇(MeOH)购自美国VWR Chemicals BDH公司。

### 1.2 AuGC/ZIF-8纳米复合材料的合成

在室温下将HAuCl_4_(20 mmol/L, 0.50 mL)、GSH(100 mmol/L, 0.15 mL)和Cys(50 mmol/L, 0.15 mL)水溶液加入至4.20 mL去离子水中混合均匀,然后加热至70 ℃,温和搅拌24 h,得到淡黄色溶液,即AuGC,于4 ℃保存备用。

将Zn(NO_3_)_2_·6H_2_O水溶液(0.50 mol/L, 2 mL)加入至2-MIM溶液(3.45 mol/L, 20 mL)中混合均匀,室温下匀速搅拌0.5 h,以16000 g离心10 min后,收集得到白色固体,用去离子水洗涤3次,于60 ℃真空干燥,得到白色粉末,即ZIF-8^[21]^。

将ZIF-8粉末(20 mg)均匀分散在AuGC溶液(5 mL)中,并匀速搅拌3 h,得到淡黄色固体,用去离子水洗涤,真空干燥,即可得到AuGC/ZIF-8纳米复合材料。

### 1.3 样品前处理

将1 mg HRP溶于0.5 mL 25 mmol/L NH_4_HCO_3_中,于95 ℃加热10 min,使蛋白质变性,冷却至室温后,加入胰蛋白酶(1∶40,质量比),于37 ℃孵育16 h,然后室温下加入2 μL甲酸终止反应。冷冻干燥后于-20 ℃保存备用。

### 1.4 糖肽富集

将100 μg AuGC/ZIF-8均匀分散在100 μL含有HRP酶解物(30 ng/μL)的上样缓冲液ACN-H_2_O-TFA(95∶4.9∶0.1, v/v/v)中,混合均匀后于37 ℃振荡孵育0.5 h。然后,用洗涤缓冲液ACN-H_2_O-FA(85∶15∶0.5, v/v/v)洗涤3次。最后,用15 μL洗脱缓冲液ACN-H_2_O-FA(30∶70∶0.5, v/v/v)振荡孵育10 min洗脱富集的糖肽。取1 μL洗脱的糖肽与1 μL 30 mg/mL DHB溶液(溶于ACN-H_2_O-TFA(70∶29∶1, v/v/v))混合均匀,进行MALDI-TOF MS分析。

### 1.5 MALDI-TOF MS条件

离子源:电喷雾电离(ESI)源;离子源温度:110 ℃;离子模式:正离子反射器模式;加速电压:20 kV;碰撞解离能量:40 eV;氮气激光:337 nm;去溶剂气体及流速:氮气,8 L/min;检测范围:*m/z* 1000~5500。采用Flex Analysis(4.0)软件对结果进行分析;选择信噪比(*S/N*)>3的质谱峰统计分析。

## 2 结果与讨论

### 2.1 AuGC/ZIF-8纳米复合材料的合成与表征

图1a为AuGC/ZIF-8纳米复合材料的合成示意图。通过GSH(或Cys)配体中的羧基与Zn^2+^之间的配位相互作用实现AuGC固定化修饰在ZIF-8表面。ZIF-8在温和条件下容易合成,其均一的多孔结构和较大的比表面积,是理想的纳米基质材料。由于GSH配体的电荷和空间稳定作用,核壳结构的AuGC纳米簇具有良好的稳定性和水溶性。GSH在合成过程中兼具还原和保护剂作用^[22]^。Cys进一步减少AuGC纳米簇表面的空间位阻(见图1b),为糖肽富集提供更多的捕获位点,从而实现高效的糖肽富集。

**图1:**
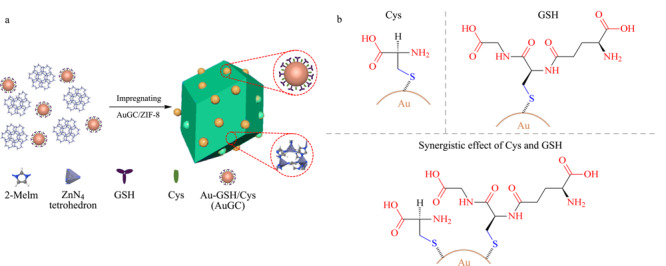
(a)AuGC/ZIF-8纳米复合材料的合成过程和(b)Cys、GSH双功能化金纳米簇

通过X射线衍射对AuGC/ZIF-8的晶体进行结构表征。结果观察到在2*θ*为011°、002°、112°、222°、134°处有典型的ZIF-8型衍射峰,表明AuGC/ZIF-8晶体结构完好^[23,24]^。利用X射线光电子能谱对AuGC/ZIF-8进一步表征,检测到Zn 2*p*、O 1*s*、C 1*s*、S 2*p*、Au 4*f*对应的峰谱。Au 4*f*的高分辨率XPS光谱结果显示,Au-S键的结合能峰值在83.9 eV(Au 4*f* 7/2)和87.5 eV(Au 4*f* 5/2)附近,与报道高度一致^[21]^。FT-IR指纹图谱中,在1500~1660和1260 (COO-)、1403 (C-N)、2550~2750 (-SH)、2900~3420(-NH_2_) cm^-1^处分别出现了吸收峰^[25,26,27]^,表明AuGC成功修饰在ZIF-8的表面上。此外,AuGC/ZIF-8比表面积为701.57 m/g,水接触角为20.82°,表明合成的纳米材料比表面积较大且具有超亲水性。

### 2.2 AuGC/ZIF-8用于糖肽富集分析

实验采用MALDI-TOF MS,考察了AuGC/ZIF-8对标准糖蛋白HRP糖肽的富集性能。首先比较了富集前和AuGC/ZIF-8富集后的糖基化肽的质谱图(见图2)。通过分析,由于信号干扰和非糖肽的抑制,富集前仅检测到一个HRP糖肽的信号(见图2a)。相比之下,富集之后显著降低了非糖肽引起的背景噪声,检测到19个糖肽峰(见图2b)。结果表明,AuGC/ZIF-8对糖肽具有显著的富集作用。富集到的糖基化肽段的详细序列信息见表1。

**图2:**
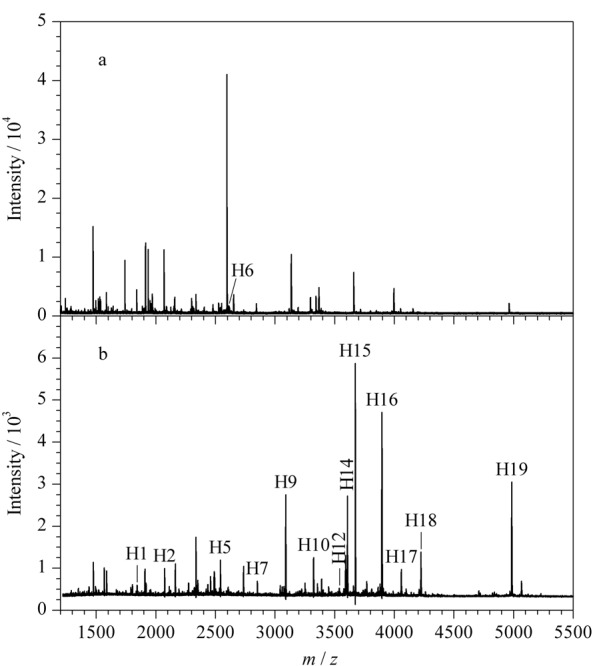
HRP酶解液(3 μg)用AuGC/ZIF-8富集 (a)前、(b)后的MALDI-TOF MS质谱图

**表 1:** 经AuGC/ZIF-8富集后HRP酶解物中糖肽的详细信息

No.	*M*_r_(*m/z*)	Glycan composition	Peptide sequence
H1	1842.841	[Xyl]1[Hex]3 [Fuc]1[HexNAc]2	NVGLN#R
H2	2073.113	[Xyl]1[Hex]3 [Fuc]1[HexNAc]2	PN#VSNIVR
H3	2273.156	[Xyl]1[Hex]2 [Fuc]1[HexNAc]2	SILLDN#TTSFR
H4	2322.100	[Hex]2 [HexNAc]2	MGN#ITPLTGTQGQIR
H5	2541.128	[Xyl]1[Hex]3[Fuc]1[HexNAc]2	SSPN#ATDTIPLVR
H6	2611.216	[Xyl]1[Hex]3[HexNAc]2	MGN#ITPLTGTQGQIR
H7	2850.382	[Fuc]1[HexNAc]1	GLIQSDQELFSSPN#ATDTIPLVR
H8	3073.335	[Fuc]1[HexNAc]1	LHFHDCFVNGCDASILLDN#TTSFR
H9	3088.337	[Xyl]1[Hex]3[Fuc]1[HexNAc] 2	GLCPLNGN#LSALVDFDLR
H10	3321.343	[Xyl]1[Hex]3[Fuc]1[HexNAc]2	QLTPTFYDNSCPN#VSNIVR
H11	3354.298	[Xyl]1[Hex]3[Fuc]1[HexNAc]2	SFAN#STQTFFNAFVEAMDR
H12	3372.248	[Xyl]1[Hex]3[Fuc]1[HexNAc]2	SFAN#STQTFFNAFVEAM*DR
H13	3537.497	[Hex]3[Fuc]1[HexNAc]2	GLIQSDQELFSSPN#ATDTIPLVR
H14	3605.486	[Xyl]1[Hex]3[Fuc]1[HexNAc]2	NQCRGLCPLNGN#LSALVDFDLR
H15	3671.579	[Xyl]1[Hex]3[Fuc]1[HexNAc]2	GLIQSDQELFSSPN#ATDTIPLVR
H16	3894.496	[Xyl]1[Hex]3[Fuc]1[HexNAc]2	LHFHDCFVNGCDASILLDN#TTSFR
H17	4056.530	[Xyl]1[Hex]3[HexNAc]2	QLTPTFYDNSC(AAVESACPR)PN#VSNIVR-H_2_O
H18	4221.659	[Xyl]1[Hex]3[Fuc]1[HexNAc]2	QLTPTFYDNSC(AAVESACPR)PN#VSNIVR
H19	4982.884	[Xyl]1[Hex]3[Fuc]1[HexNAc]2 or	LYN#FSNTGLPDPTLN#TTYLQTLR
		[Xyl]1[Hex]3[Fuc]1[HexNAc]2	

N#: *N*-linked glycosylation site.

通过分析不同AuGC/ZIF-8用量下(10、20、40、60、80、100 μg),富集到HRP特征糖肽质谱丰度的大小,考察AuGC/ZIF-8的富集能力。结果表明,AuGC/ZIF-8对糖肽的富集能力高达250 μg/mg。

考虑到复杂样品中糖蛋白的丰度较低,本研究进一步考察了基于AuGC/ZIF-8富集的MALDI-TOF MS分析方法的灵敏度。如图3a所示,分别测定了不同HRP含量下(50、3、0.3 ng/μL)该方法对糖肽的检测能力。结果表明,当HRP含量低至0.3 ng/μL时,仍然可以观察到2个较强的糖肽信号(*S/N* > 3),表明本实验方法具有较高的灵敏度,对于选择性富集复杂样品中的低丰度糖肽具有重要意义。

**图3:**
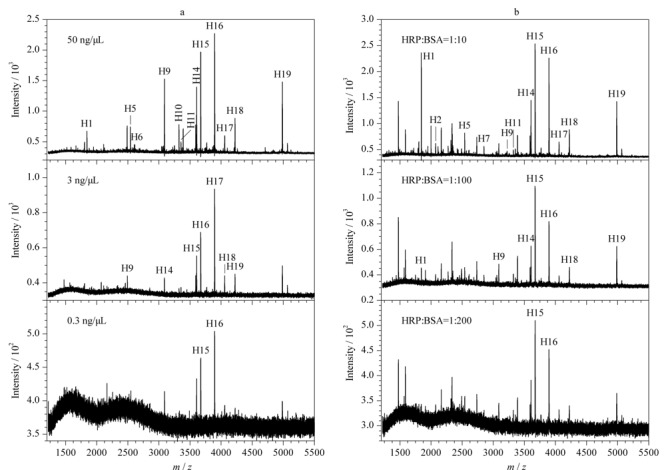
(a)不同含量HRP酶解液和(b)不同质量比的HRP和BSA酶解液通过AuGC/ZIF-8富集后的MALDI-TOF MS质谱图

为了进一步验证该方法对糖肽的选择性,配制了不同质量比的HRP和BSA的混合物(1∶10、1∶100、1∶200)进行质谱分析。结果如图3b所示,即使比例低至1∶200,经过AuGC/ZIF-8富集后,仍然可以检测到2个高信号强度的糖肽,几乎不受非糖肽的干扰,说明本实验方法对糖肽具有较强的选择性富集能力。

## 3 结论

本研究通过一种简便且低成本的方法合成了新型的双功能HILIC纳米复合材料(AuGC/ZIF-8),同时与MALDI-TOF MS联用,成功用于糖基化肽的选择性富集分析。AuGC/ZIF-8具有较大的富集能力,较高的检测灵敏度以及出色的糖肽选择性,在复杂生物样品的糖蛋白组学研究中将具有巨大的潜力。
